# Cellular and Molecular Nature of Fragmentation of Human Embryos

**DOI:** 10.3390/ijms23031349

**Published:** 2022-01-25

**Authors:** Anna Cecchele, Greta Chiara Cermisoni, Elisa Giacomini, Monica Pinna, Paola Vigano

**Affiliations:** 1 Infertility Unit, Fondazione IRCCS Ca’ Granda Ospedale Maggiore Policlinico, 20122 Milan, Italy; anna.cecchele@policlinico.mi.it (A.C.); monica.pinna@policlinico.mi.it (M.P.); 2 Obstetrics and Gynaecology Unit, IRCCS San Raffaele Scientific Institute, 20132 Milan, Italy; cermisoni.greta@hsr.it; 3 Reproductive Sciences Laboratory, Obstetrics and Gynaecology Unit, IRCCS San Raffaele Scientific Institute, 20132 Milan, Italy; giacomini.elisa@hsr.it

**Keywords:** embryo, fragmentation, micronuclei, vesicles, apoptosis

## Abstract

Embryo fragmentation represents a phenomenon generally characterized by the presence of membrane-bound extracellular cytoplasm into the perivitelline space. Recent evidence supports the cellular and molecular heterogeneity of embryo fragments. In this narrative review, we described the different embryo fragment-like cellular structures in their morphology, molecular content, and supposed function and have reported the proposed theories on their origin over the years. We identified articles related to characterization of embryo fragmentation with a specific literature search string. The occurrence of embryo fragmentation has been related to various mechanisms, of which the most studied are apoptotic cell death, membrane compartmentalization of altered DNA, cytoskeletal disorders, and vesicle formation. These phenomena are thought to result in the extrusion of entire blastomeres, release of apoptotic bodies and other vesicles, and micronuclei formation. Different patterns of fragmentation may have different etiologies and effects on embryo competence. Removal of fragments from the embryo before embryo transfer with the aim to improve implantation potential should be reconsidered on the basis of the present observations

## 1. Introduction

Embryo fragmentation represents a phenomenon generally characterized by the presence of membrane-bound extracellular cytoplasm into the perivitelline space. In vitro, human embryo fragmentation has been reported since the 1980s [1], but it has been described also in human embryos conceived in vivo [2], indicating that it is not an artifact of the in vitro culture. Many diverse terms have been used to refer to these cytoplasmic fragments, including corpse, cytoplasmic pinching, micronuclei, debris, and shedding microvesicles. Highlights from the current literature support the cellular and molecular heterogeneity of embryo fragments: they can vary in size, kinetics, and organelle and molecular content [3]. Importantly, during assisted reproduction technology (ART) procedures, fragmentation and cell debris are considered important prognostic factors in the static morphologic assessment of human embryo quality, along with cell number, size, and symmetry. In this context, the presence of cytoplasmic fragments is suggestive of a poor prognosis embryo development and poor ART outcomes. On the basis of this idea but without strong supporting backgrounds, some groups have proposed to remove these cellular structures from the embryos before the transfer [4]. More recently, time-lapse microscopy (TLM) documented that cellular fragments can be extruded or reabsorbed into blastomeres, highlighting a dynamism in the process [5]. 

Thus, the impact of fragment-like entities on the embryo developmental competence remains to be clearly elucidated. Most importantly, novel information on this topic suggests that fragments may have different origins with a possible range of effect on embryo competence. In this review, we intended to collate the older and more modern literature regarding the origins of cytoplasm fragmentation in the various steps of pre-implantation embryo development. In addition, we described the alternative theories on the origins of these phenomena over the years, underling strengths and limitations. We pursued the following aims: (i) description of the different embryo fragment-like entities in their morphology, molecular content, and supposed function, and (ii) reporting the proposed theories on the origin over the years. 

## 2. Results

### 2.1. Different Fragment-like Cellular Structures: Characterization, Timing, and Cargo

- Fragment size

**Figure 1:**
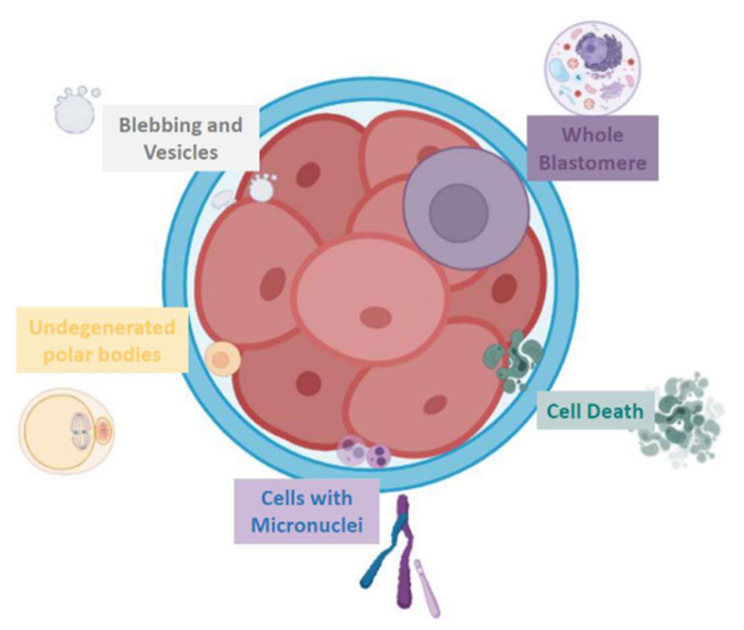
Schematic representation of the various fragment-like cellular structures detected in embryos.

Embryo fragments are heterogeneous in size: they can vary from normal-size blastomeres to simple cellular debris. Johansson et al. classified 44 cleavage embryos according to fragment size: entities smaller than 45 µm in day 2 and smaller than 40 µm in day 3, respectively, have been considered as anucleated cytoplasmic fragments, while larger structures as blastomeres [6]. In addition, human embryo can naturally release extracellular vesicles (EVs) that, on the basis of cellular origin, size, and release mechanism, can be categorized into exosomes (30–150 nm in diameter) [7], microvesicles (50–1000 nm) [8], and apoptotic bodies (50 nm–5 µm) [9,10,11,12] (Figure 1).

- Timing of cytoplasmic fragment formation

**Figure 2:**
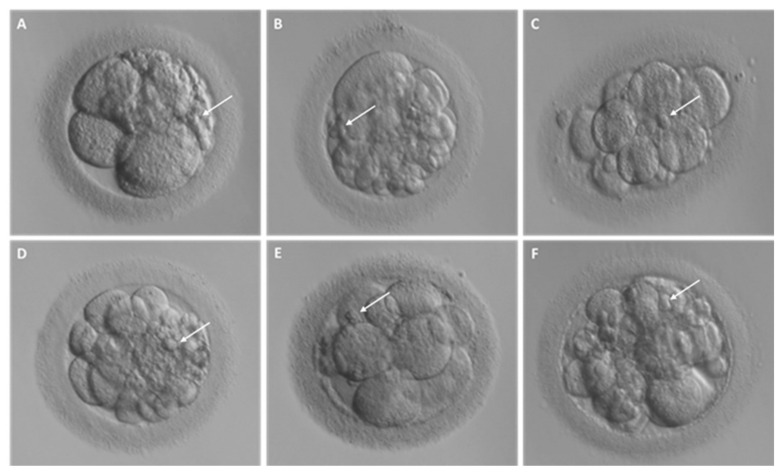
Different degrees of fragmentation in cleavage stage human embryos. (**A**–**F**) Representative images of day 3 embryos characterized by cellular fragments of different sizes and positions. For each embryo, arrows indicate a representative cellular fragment.

**Figure 3:**
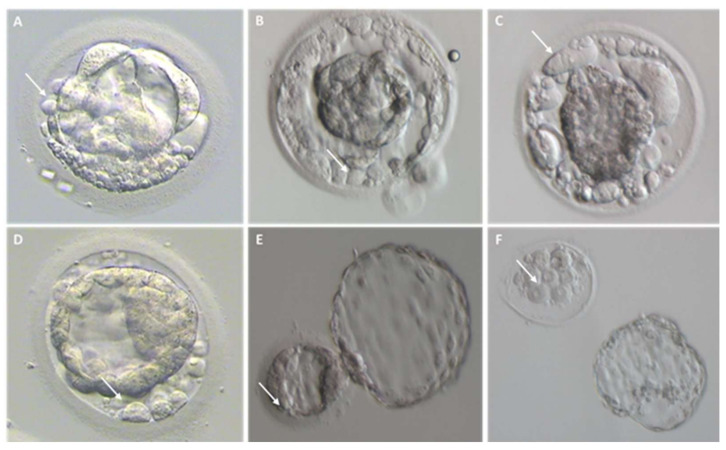
Cellular debris/fragments in the zona pellucida of day 5, 6, and 7 blastocysts. For each embryo, arrows indicate a representative cellular fragment or blastomere excluded upon blastocyst formation. (**A**–**D**) Representative images of collapsing blastocysts presenting different degree of fragmentation; (**E**) hatching blastocyst characterized by several cellular fragments within the zona pellucida; (**F**) hatched blastocyst on the right and its original zona pellucida containing leftovers of cell debris on the left.

**Figure 4:**
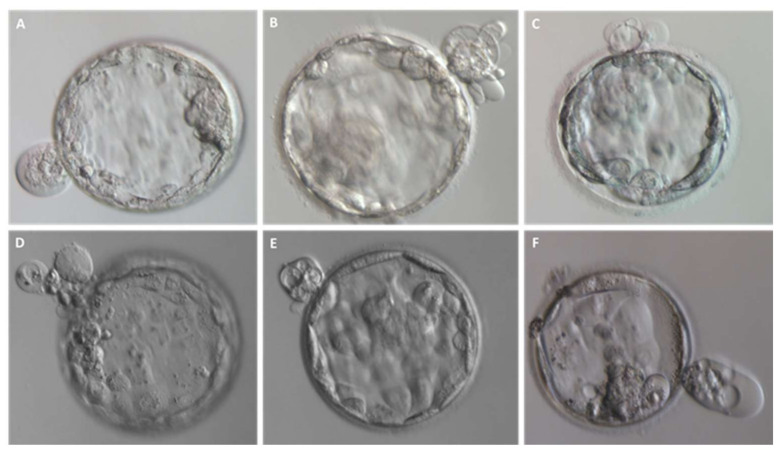
Entire blastomeres are excluded upon blastocyst formation. (**A**–**F**) Representative images of expanded blastocysts expelling one or more cells.

Fragmentation may occur from the first embryo division of pre-implantation development when the maternal genome drives the development; this phenomenon has been initially suggested to be less common after the embryonic genome activation [13]. Interestingly, representative time-lapse frames exhibited dissimilar temporal and spatial patterns of fragmentation among various steps of pre-implantation embryo development [3]. Fragments formed in some embryos during the pronuclear or early cleavage stages but were no longer detectable at later stages. For other embryos, some of the cellular fragments occurring during early cleavage were still detectable in blastomeres during late cleavage [3] (Figure 2). Direct evidence of the dynamic nature of the phenomena was subsequently reported. Handarson and colleagues described by time-lapse sequence imaging the internalization/reabsorption of cellular fragments into neighboring blastomeres, while others disappear, leaving behind only debris [5]. A more recent suggestion is that an embryo can be capable of excluding any unwanted cell and/or cellular fragment from the remaining viable cells during the morula-to-blastocyst transition [14] (Figure 3). The complete exclusion of fragments or entire blastomeres can be observed in compacted embryos, morulae and blastocysts (Figure 4). Commonly, fragments do not take part in the blastocyst formation. This phenomenon is usually not very visible when the blastocoel inside blastocyst increases causing the progressive thinning of the zona pellucida (ZP). It is more visible when the blastocyst shows collapse or contraction episodes: some cells or fragments or debris become clearly visible in the perivitelline space (Figure 3). 

Similarly, the release of human embryo-derived EVs has been reported from the zygote stage up to expanded blastocyst stage [15,16]. Indeed, EVs were detected in the zona pellucida of human zygotes and in embryo conditioned culture media, but not in the ZP of metaphase II oocytes, suggesting that EV release begins shortly after fertilization [16].

- Fragment cargo

The cargo of embryo fragments (from entire blastomere up to EVs) may contain nucleic acids, proteins, lipids, chromosomes, and entire organelles. Organelles as vacuoles, large mitochondria, vesicle complexes, and lysosomes may be sequestered into fragments [17,18]. Entire or portions of chromosomes, sequestered during fragmentation, can originate from either the mother or the father [19,20]. Evidence reveals that a preferential sequestering of particular chromosomes is unlikely. Chromosomal fragment size was found to range from 6 to 85 Mb [20]. 

#### The Different Origins Proposed for Embryo Fragment-like Entities

1. Extruded blastomeres

Thanks to the use of TLM that allows a deeper observation of the events underling embryo development, blastomere exclusion has been observed both in morulae and blastocysts of several mammalian species (i.e., humans, rhesus macaques, cattle, and mice) [21,22,23,24,25,26,27,28,29] (Figure 3). Blastomeres could be excluded during the first phases of embryo compaction, while others could be extruded from the compacted morula after a transient involvement during this process [14]. Lagalla et al. retrospectively evaluated 791 embryos obtained in 145 ART cycles by time-lapse morphokinetics analysis. Array-CGH analyses performed on both trophoectoderm cells and those excluded during morula compaction demonstrated that the latter have a higher incidence of aneuploidies as compared to the former ones. Those extruded cells are unable to flatten and establish tight intercellular contacts. Several factors may be involved in the failure of the compaction process: an abnormal formation of tight junctions [29] or the inability to express proteins involved in cell adhesion [13]. The frequency of this phenomenon is not a well-documented process, but it is clearly known that pre-implantation embryos tolerate it well.

2. Chromosome-containing micronuclei

Although initially embryonic fragments were considered as anucleate cytoplasmic components, subsequently, the presence of nuclear DNA started to be demonstrated [19,30,31]. By performing immunofluorescence analysis using antibodies against the centromere protein-A (CENP-A) and the nuclear envelope marker, LAMIN-B1, Chavez and colleagues demonstrated the presence of micronuclei in cleavage stage human embryos, suggesting their formation could be a mechanism adopted by embryos to sequester mis-segregated chromosome [19]. Micronuclei are extra-nuclear bodies containing damaged chromosome fragments and/or whole chromosomes that were not incorporated into the nucleus after cell division. They originate from acentric chromatid/chromosome fragments or from entire chromatids/chromosomes that are not correctly included in the main nucleus during the telophase [32]. They are, instead, enwrapped by the nuclear membrane, acquiring the structure of the daughter nucleus, even though smaller in size [33,34]. Fragments containing micronuclei can be reabsorbed by the embryo and fused with neighboring blastomeres, thus possibly resulting in the correction of aneuploid blastomeres or in altering the correct ploidy status if they fused with euploid blastomeres. Thus, this process can have a positive effect since, if tolerated, it can influence karyotype evolution in species, or the result may be deleterious, leading to additional genetic mutation [19].

3. Apoptotic bodies

Since the 1980s, a role of apoptosis process in pre-implantation embryo development has been suggested. The first report demonstrating the presence of ultrastructural features associated with degenerating cells and with a micro-pinocytotic activity in the inner cell mass (ICM) cells of viable/hatched human blastocysts was published in 1982 [35,36]. This latter study confirmed previously published results in primate models [36], suggesting a physiological role of cell death process in ICM development. Nevertheless, the pioneers of ART realized that both viable and arrested embryos may contain a proportion of cytoplasmic apoptotic fragments, suggesting that cellular apoptosis could play a role also in embryonic arrest. A detailed description of the controversial findings on the apoptotic phenomena associated with embryo fragmentation is reported in a subsequent paragraph (Section 2.2 point 1.). A distinctive morphological change of apoptosis is the blebbing and the consequent apoptotic bodies formation. These membrane-bound vesicles contain cytoplasm, organelles, and nuclear fragments, and they are released into the extracellular space. After the release, apoptotic bodies are usually phagocytosed and degraded/digested by professional phagocytes (such as macrophages) or non-professional phagocytes (such as epithelial cells) [37]. The phagocytic competence of early embryonic cells has been proposed following the observation of in vitro internalization of fluorescence microspheres in trophectoderm cells of human blastocysts after overnight co-culture evaluated by transmission electron microscopy (TEM) and fluorescence microscopy [38]. Li et al. did not detect phagocytic activity in the ICM, and an increased ability in blastocysts after 6 days of culture was observed compared to faster ones. However, the authors did not investigate the molecular mediators of phenomenon. Phagocytosis is a very specific process, characterized by several successive steps and mediated by fine interactions between cell surface ligands and cell surface receptors. It is also true that Pisko and colleagues supported this idea in a mouse model where embryonic cells had all the key molecules necessary for the recognition and digestion of damaged blastomeres, undertaking the clearance of the majority of cellular debris in blastocysts [39]. Despite the above assumptions, many cellular fragments mostly persist in the blastocoel and in the perivitelline space, suggesting that other mechanisms may be responsible for fragmentation and debris formation in pre-implantation embryos.

4. Persisting polar bodies

Polar bodies (PBs) are the byproduct of the oocyte meiotic cell divisions. They are small cytoplasmic blebs containing haploid genetic material plus a small amount of cytoplasmic organelles. Generally, they undergo apoptosis in 17–24 h after formation [40]. Evidence suggests that persisting PBs in embryos at the blastocyst stage can give rise to cellular fragments in the sub-zonal space [41]. Ottolini and colleagues performed a genetic analysis of a slow developing embryo; they analyzed the trophectoderm and the excluded cell fragments. Results obtained from the karyotyping of the fragments (47,XX,+19) and of the trophectoderm sample (46,XY) were non-concordant. In order to investigate deeper the cause of these results, DNA fingerprinting analyses using a panel of informative short tandem repeats markers and amelogenin were performed in all the samples. Subsequently, samples were also analyzed for karyomapping. The fragments demonstrated the absence of any paternal alleles and the presence of only a single maternal allele at each locus. The karyomapping revealed that the DNA amplified from fragments was exclusively that of the second polar body corresponding to the fertilized oocyte that gave rise to the embryo from which the trophectoderm had been biopsied [42]. This study demonstrated that a fraction of fragments may derive from the second PB.

5. Extracellular Vesicles

In 2019, Vyas and collaborators demonstrated that EVs could be released from all stages of pre-implantation embryos including 1-cell zygotes, cleavage embryos (2-cell, 4-cell, and 8–10-cell), morulae, and blastocysts. EVs were also detected throughout the ZP from the inner to its external surface, suggesting the capability of these vesicles to pass through ZP. In the same year, Battaglia and colleagues reported the presence of EVs in human blastocoel fluid [42]. Some studied confirmed that embryo-derived EVs are present in embryo-conditioned culture media of both day 3 and day 5 human pre-implantation embryos and, in both cases, with a diameter between 50 and 200 nm, consistent with exosome and microvesicle size [9,16]. Their specific molecular cargo (OCT4 and NANOG gene transcripts, HLA-G protein) suggests that they can arise from both ICM and trophectoderm compartment [9]. Unlike other cytoplasmic fragments, EVs have been shown to act as mediators of active cell-to-cell communication by packaging and transferring molecules from one cell to another both locally and remotely [43].

6. Others

- Mitochondria

Fragmented embryos displayed a different organization of mitochondrial distribution: a higher concentration of mitochondria has been observed in the center rather than in the periphery of blastomeres in fragmented embryos as compared to non-fragmented ones [44]. This pattern could be linked to reduced adenosine triphosphate (ATP) content and reduced developmental potential [45] that can ultimately result in the disruption of the membrane with subsequent cellular lysis caused by disruption of the ion pump function [3].

- Perivitelline threads

Perivitelline threads (PVTs) (also defined with the term of trans-zonal projections) are thin filaments that extend across the perivitelline space connecting the ZP with the oolemma or with the blastomere membrane. Their origin and nature are not clear. A theory linked their formation to the corona radiata; indeed, corona radiata cells are characterized by projections that can traverse the ZP with a role in the communication with the oolemma before ovulation [46,47]. After the luteinizing hormone surge, these projections are withdrawn and are thought not to persist beyond the meiotic reactivation stage. Nevertheless, observations during intracytoplasmic sperm injection, demonstrated that remnants of the projections of corona radiata persist, thus resulting in the formation of PVT [48]. Derrick and colleagues demonstrated an association between the presence of PVTs and embryo fragments. Analyzing 525 blastocysts, the authors found that 77% of them were characterized by the presence of PVTs, most appearing at the 2-cell stage (98% of the cases). Almost all of the embryos characterized by PVTs presented fragments (98%). Conversely, fragmentation was significantly less frequently observed in embryos without PVTs [48]. During the first mitotic division, a tight adherence between the PVT and the membrane may cause a strain during movement of the cells, causing fragments to form where there are already some weaknesses, thus explaining the link between PVTs and the generation of fragments [49]. Notably, there is no evidence of a relationship between PVTs and implantation potential (implanted embryos with PVT vs. without PVT: 25 vs. 29%) or with the ploidy status (euploid embryos with PVT vs. without PVT: 40 vs. 49%), suggesting no significant relationship between PVTs and embryo developmental potential [48]. 

### 2.2. Theories on the Origins over the Years

The precise mechanism(s) by which embryo fragmentation occur remains to be clarified. The cellular machineries involved in fragment-like entities formation and release could be unique or various (depending on type of cellular content or the timing of formation). Nevertheless, several hypotheses on the origin of fragments have been proposed, including apoptotic cell death, the effect of reactive oxygen species, cytoskeletal disorders, vesicles and micronuclei formation. All these theories might be valid. In addition, the frequency of this phenomenon in humans is also unknown. Notably, as a general idea, it has to be underlined that, in recent years, the interest in chromosome abnormalities and chromosomal mosaicism in human pre-implantation embryos has increased. As a consequence, the presence of whole chromosomal abnormalities or aneuploidy has been considered to be a primary determinant of whether an embryo arrests or reaches the blastocyst stage [50]. Thanks to the use of high-resolution techniques, it has been estimated that between 50% and 80% of cleavage stage human embryos contain more than one aneuploid cell [10,51,52,53,54,55]. Because of the lack of cell cycle check points during blastulation, different types of mosaicism (i.e., aneuploid/diploid mosaicism and complex aneuploid mosaicism) have been frequently found in embryos [55,56]. Despite the majority of chromosomal errors not being corrected, there are several lines of evidence supporting the existence of “embryo self-corrective” mechanisms that are involved in the extrusion of aneuploid cells during embryo development [57,58]. These mechanisms are thought to be the results of multipolar divisions, blastomere exclusion, and cellular fragmentation. The hypothesis that embryo fragmentation could be a tool of regulation and maintenance of cellular homeostasis in human embryo was supported by reported associations between extensive fragmentation and chromosomal abnormalities [59,60,61,62]. 

However, different mechanisms were studied over the years linking embryo homeostasis and the release of fragments, uneven cells, or debris and they are described below.

1. Apoptotic cell death

Apoptosis involves redistribution of membrane phospholipids within the lipid bilayer, nuclear fragmentation, cytoplasmic shrinkage, and plasma membrane protuberans known as blebs [63,64]. One of the early events of the apoptotic process, before the loss of cell membrane integrity, is the phospholipid phosphatidylserine translocation to the outer leaflet of the membrane bilayer [65]. Phospholipid phosphatidylserine externalization can be easily detected using annexin V, a phosphatidylserine-binding protein. This apoptosis stage is strongly associated with chromatin condensation events on the inner nuclear membrane [66], a process that can be detected by labelling DNA with specific fluorochromes such as propidium iodide (PI) and 4′,6-diamidino-2-phenylindole (DAPI). Another important feature of late phase apoptosis is the DNA fragmentation that can be detected by the terminal deoxynucleotidyl transferase dUTP nick end labeling (TUNEL) assay [67]. Correlation of cellular fragmentation with apoptosis in fragmented or normally developing human cleavage stage embryos represents a controversial finding. Yang and colleagues reported a TUNEL signal in most fragmented cleavage stage embryos (from the two-cell to eight-cell stage) but not in non-fragmented ones [68]. Antczak and Van Blerkom reported no TUNEL signal or annexin V fluorescence in both fragments and in intact blastomeres of living fragmented embryos between 2- and 8-cell stages [69]. Levy and colleagues reported increased annexin V staining and TUNEL assay labelling in arrested and fragmented day 2 embryos but no annexin V staining in cleavage stage embryos normally developing after thawing [70]. Jurisicova and colleagues also proposed cellular fragmentation as a consequence of embryo programmed cell death of blastomeres in human cleavage embryos arrested at different stage of development. Several cellular fragments containing organelles and condensed chromatin within the ZP, associated with apoptosis markers (TUNEL staining) as well as caspase-2 and caspase-3 mRNA expression, were observed in these embryos [71,72]. Studies investigating apoptosis pathways reported that some markers such as BAX and BCL (mRNA and proteins) were even expressed from unfertilized oocyte, while others such as PDCD5, BAD (mRNA), caspases, and Harakiri were expressed mainly at the blastocyst stage [71]. Martinez and colleagues frequently observed positivity for caspase activity in fragments but rarely in normal blastomeres of arrested embryos. No differences were detected in the proportion of caspase-positive cellular fragments between 2-cell and 12-cell stage embryos, thus before and after embryonic gene activation [73]. In 2001, with an integrated approach between retrospective data and mathematical modeling, apoptosis episodes were demonstrated from morula to blastocyst stage in viable embryos of good morphology [74]. Hardy and colleagues assessed morphological and biochemical markers of apoptosis in fixed zona-free embryos at different developmental stages until blastocyst stage by using confocal microscopy. Nuclear morphology was evaluated after treatment of samples with DAPI, and fragmented DNA detection was evaluated by TUNEL. The levels of TUNEL-labelled cells substantially increased at blastocyst stage, while apoptosis markers were absent in cleavage stage embryos. The appearance of apoptotic markers has been associated with important steps of pre-implantation embryogenesis: the activation of the embryonic genome, the development of gap junctions, and the maturation of mitochondria. Cell–cell communication via gap-junctions, in particular, rarely present in cleavage stage embryos, was supported as a molecular requirement for apoptotic signal propagation [75,76]. In line with the idea that selection/correction of aneuploidies are one of the mechanisms for fragmentation of the embryos, Santos and colleagues observed aneuploid blastomeres leaving the blastocyst following the activation of apoptotic pathways [77,78]. Recently, also, the blastocoel fluid was analyzed for the presence of apoptosis markers [43,79]. Caspase-3 protease activity has been detected in this compartment, supporting the idea that a fraction of molecules in blastocoel fluid are products of apoptotic embryonic cells [80]. In general, however, several aspects still need to be elucidated, i.e., factors affecting blastomere apoptosis and the entity of the phenomenon in the human pre-implantation embryo at different stages.

2. Reactive oxygen species effect

Generated during the physiological consumption of oxygen, reactive oxygen species (ROS) can be the product of the embryo metabolism, but they may also originate from embryo surroundings [81]. High levels of ROS along with an imbalanced formation of antioxidants is thought to result in oxidative stress, resulting in suboptimal embryos competence [82,83]. Indeed, differently from what occurs in vivo, in which the presence of antioxidants or antioxidative enzymes in the fluid and epithelium of oviduct protects the embryo from ROS, in an in vitro culture system, levels of these compound have been demonstrated to inversely correlate to embryo developmental competence [81,84,85]. Interestingly, several studies have reported a positive correlation between ROS levels in the spent medium and the fragmentation rate in human embryos at cleavage and blastocyst stage [84,86]. Thanks to the use of imaging techniques, such as TEM and other fluorescence assays, ROS have been detected at a higher concentration in embryos with a higher rate of cellular fragmentation [68]. While a certain amount of ROS may benefit the embryo development, as mitochondrial oxidative phosphorylation is an efficient way to produce ATP but at a cost of ROS generation, elevated ROS levels have harmful effects, including DNA damage and alteration of most types of cellular molecules [86]. Nevertheless, a recent study reported a lack of association between ROS levels in media of cultured individually embryos (as evaluated by a chemiluminescence assay using luminol) and embryonic development or high embryo fragmentation [87].

3. Membrane compartmentalization of DNA

As mentioned above, micronuclei have been detected in cleavage stage human embryos as a whole chromosome or a fragment of a chromosome that is not incorporated into one of the daughter nuclei during cell division. There is no evidence of a preferential association of aneuploidy with a subtype of chromosomes: both large and small chromosomes can be sequestered [88]. In addition, mis-segregated chromosomes and chromatid fragments encapsulated within micronuclei are dynamic entities: they may persist, rejoin the primary nucleus, or might be definitely eliminated from the embryo, in line with the theory of embryo “self-correction”. Moreover, chromosomes can undergo a specific phenomenon of “chromosome pulverization”, known as chromothripsis, that allows the reduction of one or a few chromosomal fragments into many pieces, randomly reassembled in one unique cellular event during a single-cell division [59]. Not all the fragments are characterized by the presence of sequestered micronuclei; thus, the rearrangement of fragments does not always result in the alteration of the ploidy [5,19,89].

4. Abnormal cytokinesis and cytoskeletal disorder

It is well documented that embryos with abnormal duration of cell cycles and cytokinesis (generally, with a delayed first mitosis, an earlier start of the second mitosis, and a longer duration of the third mitosis) are more likely to be fragmented [13]. An incorrect cell cycle may result in genomic alterations because the cell does not have enough time to correct any eventual error during DNA replication. Alikani and colleagues reported that loss of interplay between the spindle complex and cortical microfilaments was associated with blebs and cellular fragments formation. In addition, the authors demonstrated that treatment with cytokinesis inhibitors prevented cytokinesis as well as fragment formation, supporting a cause–effect relationship [13]. Stensen and colleagues also linked the rate of embryo fragmentation with the duration of meiotic process. A delay in the oocyte meiotic division (formation of the meiotic spindle 36.2 h after human chorionic gonadotropin injection) was associated with higher fragmentation rates [50–100%] in resultant embryos [90]. The reason underlying this observation may be related to cell cycle defects implicated in oocyte aneuploidy involving alterations in chromosome pairing, recombination, and spindle assembly, resulting in a delayed meiotic cell cycle [91,92,93]. No correlation, instead, was found between fragmentation and other spindle characteristics (i.e., a delay in its formation and the angle calculated between the first polar body and the meiotic spindle). Lastly, according to the same group, the process of fragmentation was more pronounced during the early phases of cell division, when the maternal genome is still active. After the activation of the embryonic genome, the tendency of human blastomeres to fragment would be lost. Extruded blastomeres from these embryos would express maternal instead of embryonic transcripts, during an inappropriate timing for the developmental stage [90].

5. Extracellular vesicle formation

Human embryos can secrete EVs in their culture media that can be easily taken up by endometrial cells [15]. They are formed through multiple biogenetic pathways: (i) Exosomes are generated from the endosomal system by the formation of late endosomes, which are formed by inward budding of the multivesicular body (MVB) membrane. Invagination of late endosomal membranes results in the formation of intraluminal vesicles (ILVs) within large MVBs [94]. In the next step, MVBs have two fates: most ILVs are released into the extracellular space upon fusion with the plasma membrane or, alternatively, these components are trafficked to lysosomes for degradation [95,96]. (ii) Microvesicles, instead, are formed through the outward budding and fission from plasma membranes. In contrast to exosome formation, the secretion of microvesicles requires the lipid microdomains at the membrane and a reorganization of the actin–myosin cytoskeletal network [97,98]. A possible association between the EV quantity in the spent culture media and embryo quality and competence has been suggested [99,100,101]. Specifically, fewer EVs have been reported in spent culture media of embryos leading to successful pregnancy than in those who failed, suggesting that a good quality and competent embryo releases different amounts/types of EVs compared to a low-quality embryo [102,103,104,105].

## 3. Discussion

Fragmentation is a common feature of the early development of human embryos. During ART procedures, multiple clusters of fragments can be frequently observed in in vitro developing embryos. However, the causes of this cellular phenomenon and its impact on developmental competence are not so clear. Notably, the literature on this topic is for the most part dated on one hand, and, on the other hand, refers to studies far from the ART procedures [19,20,73]. The phenomenon may be the results of intrinsic processes in cleaving embryos or may be the results of external factors [106,107]. The consequence is that fragmentation can affect none, some, or all embryos, demonstrating that this phenomenon is both embryo- and patient-specific. A critical point of discussion focuses on the impact of fragment-like entities on the embryo developmental competence. A consistent body of literature linked the rate of fragmentation with a lower embryo developmental potential and implantation rate [24,32,74,108,109]. This observation could be explained by the theory supporting an association between fragment-like entities and chromosomal abnormalities. Aberrant or exceeded chromosomes may result in the formation of DNA-containing micronuclei that ultimately may lead to the inactivation of the embryonic genome and the subsequent block of embryo development [31]. On the other hand, a reduced embryo developmental potential may be explained also by those theories supporting the anucleate cytoplasmic nature of embryo cellular fragments. The loss of a large volume of cytoplasm may be detrimental to embryo potential by depleting blastocyst of essential organelles (e.g., mitochondria), mRNAs, and proteins, resulting in an early block during embryo development. More specifically, the loss of a large volume of cytoplasm may be related with the reduction of important regulatory proteins for embryogenesis (e.g., leptin, signal transducer and activator of transcription 3, BAX, Bcl-x, transforming growth factor beta 2, vascular endothelial growth factor, c-kit, and epidermal growth factor receptor) [69]. In addition, the presence of large cytoplasmic cellular fragments may also have an impact in the spatial arrangement of the blastomere in the context of ICM and trophectoderm of the blastocyst [24,110]. Indeed, they can cause apoptosis or the loss of a significant volume of cytoplasm, limiting the rate of blastomere cleavage because of the distortion of the blastomere division planes, leading to abnormal compaction, cavitation and blastocyst formation.

On the basis of these observations, cosmetic embryo microsurgery in terms of removal of fragments and coarse granulation from the embryo before embryo transfer has been suggested to improve cell division and implantation potential [4,24,32]. Sordia-Hernandez and colleagues reported results in line with this idea, but they also observed a higher rate of abortions in the group of patients who had defragmented embryos transferred. The relationship between micromanipulation and abortion is controversial and could be related to the fact that fragmented embryos could be genetically abnormal, or they could suffer important structural damage when fragment aspiration is performed [111,112]. Taken together, positive implantation outcomes after microsurgical fragment removal could be the result of the restoration of spatial relationship of cell-to-cell contacts disturbed by fragments. In addition, this procedure may prevent secondary degeneration of adjacent cells caused by debris [24].

In contrast, on the basis of the other theories presented, the removal of fragments could have a negative effect on the implantation embryo potential. In the study by Halvaei et al., larger cytoplasmic fragments were characterized by the presence of sequestered functional cellular organelles, particularly mitochondria capable of generating ATP [113]. The effect of their removal could be detrimental since it could prevent surrounding blastomeres of important organelles and of their enzymatic activity. Moreover, the same study suggested that it is not beneficial to proceed with the removal neither on embryos with a fragmentation rate between 0 and 10%, nor on embryos with more than 35% of cellular fragments. Indeed, the former embryos displayed an implantation rate very similar to those of high-grade embryos, while the removal of cytoplasmic fragments from the others may lead to an amelioration only from a morphological point of view [18]. Lastly, cellular fragments could be indicative of chromosomal problems, and therefore their removal might not improve the potential of many severely fragmented embryos.

## 4. Materials and Methods

A search in PUBMED for the peer-reviewed papers published in English from 1970 through January 2021 was conducted to identify articles related to characterization of embryo fragmentation or cellular fragments and description of the kinetic of cell extrusion/exclusion phenomena. The search string used was (embryo* OR blastocyst) AND (fragment* OR corpse OR vesicle) NOT (sperm DNA fragmentation OR sperm fragmentation). Animal models were excluded from the study.

## 5. Conclusions

It is beyond any doubt that, in the near future, a wide panel of the investigations from the fields of experimental and applied embryology should have been targeted at the precisely identifying and comprehensively exploring a broad spectrum of morphological, ultrastructural, biochemical, and molecular determinants responsible for increased incidence of the processes leading to initiation and progression of embryo fragmentation. The thorough characterization of the aforementioned determinants seems to be strongly justified in order to recognize the highly predictable biomarkers related to diminishments in not only molecular quality parameters, but also the extracorporeal and peri-implantation developmental capabilities of the ex vivo produced embryos created by such modern ARTs as in vitro fertilization and intracytoplasmic sperm injection in humans and other mammalian species and somatic cell nuclear transfer (SCNT)-mediated cloning in other mammalian species [114,115,116,117,118,119]. 

Different patterns of fragmentation may have different etiologies and effects on embryo competence. Removal of fragments from the embryo before embryo transfer with the aim to improve implantation potential should be reconsidered on the basis of the present observations.

## Abbreviations

ART	assisted reproduction technology
CENP-A	centromere protein-A
DAPI	4′,6-diamidino-2-phenylindole
EV	extracellular vesicles
ICM	inner cell mass
ILV	intraluminal vesicle
MVB	multivesicular body
PB	polar body
PI	propidium iodide
PVT	perivitelline thread
ROS	reactive oxygen species
SCNT	somatic cell nuclear transfer
TEM	transmission electron microscopy
TLM	time-lapse microscopy
TUNEL	terminal deoxynucleotidyl transferase dUTP nick end labeling
ZP	zona pellucida
